# Volatile Organic Metabolites Identify Patients with Mesangial Proliferative Glomerulonephritis, IgA Nephropathy and Normal Controls

**DOI:** 10.1038/srep14744

**Published:** 2015-10-07

**Authors:** Changsong Wang, Yue Feng, Mingao Wang, Xin Pi, Hongshuang Tong, Yue Wang, Lin Zhu, Enyou Li

**Affiliations:** 1 Department of Anesthesiology, the First Affiliated Hospital of Harbin Medical University, Harbin, China; 2 Department of Nephrology, the First Affiliated Hospital of Harbin Medical University, Harbin, China

## Abstract

Urinary volatile organic compounds (VOCs) analysis for kidney diseases has attracted a large amount of scientific interest recently, and urinary metabolite analysis has already been applied to many diseases. Urine was collected from 15 mesangial proliferative glomerulonephritis (MsPGN) patients, 21 IgA nephropathy (IgAN) patients and 15 healthy controls. Solid phase microextraction–chromatography– mass spectrometry (SPME-GC-MS) was used to analyse the urinary metabolites. The statistical methods principal component analysis (PCA) and orthogonal partial least-squares discriminant analysis (OPLSDA) were performed to process the final data. Five metabolites were significantly greater in the group of MsPGN patients than in the normal control group (P < 0.05) while three metabolites were found at increased levels in the group of IgAN patients compared with the normal controls (P < 0.05). In addition, five metabolites were significantly increased in the group of IgAN patients compared with the MsPGN patients (P < 0.05). These five metabolites may be specific biomarkers for distinguishing between MsPGN and IgAN. The analysis of urinary VOCs appears to have potential clinical applications as a diagnostic tool.

Mesangial proliferative glomerulonephritis (MsPGN) has been recognized based on light microscopic findings and immunofluorescence studies with immunoglobulin and C3 complement in glomerular mesangial area^1^. MsPGN can be classified into two categories, one is IgA nephropathy (IgAN), the other is non-IgA nephropathy (non-IgAN), i.e. MsPGN^1^,^2^. Recently, MsPGN patients are treated with corticosteroid drugs basically. Combined drugs of corticosteroid with immunosuppressant are commonly used for IgAN patients. Therefore, to determine the appropriate clinical course and a patient’s individualized treatment plan, a definitive diagnosis of the disease is important.

The current gold standard for pathological diagnosis is renal biopsy because of its specificity. Based on the renal biopsy and light microscopy and immunofluorescence studies, a clear disease classification can be diagnosed, and the appropriate treatment scheme can be determined. However, a renal biopsy is invasive and has poor repeatability with a risk of significant complications, particularly in patients who have bleeding tendency or skin infections on the flank. Thus, a renal biopsy may be contraindicated for certain high risk patients and is often refused by patients^3^,^4^.

In recent years, metabolomics has often been used as a promising technique in disease diagnosis. Biomarkers in urine, serum, saliva or plasma samples have been used to understand the metabolic changes in externally affected biological systems over time for a variety of diseases^5^. Among these samples, urine is a complex biofluid with a rich metabolite composition that reflects various metabolic processes in the organism. As a result of its ease of collection, it has been widely used in metabolomic studies to investigate pathological conditions, such as inborn errors of metabolism, diabetes, and different types of cancer^6^. For instance, urinary metabolomic studies have been applied to breast^7^, colorectal^8^, esophageal cancer^9^, pancreatic ductal adenocarcinoma^10^ and liver cancers^11^. Koichi Matsumura *et al*.^12^ demonstrated that urinary volatile organic compounds (VOCs) can assist in the diagnosis of lung cancer. The kidney is the organ in which urine is produced, concentrated, reabsorbed and excreted. Thus, when the renal system is impaired, VOCs in the urine may indicate corresponding changes. Many studies have validated the potential of VOCs to serve as a basis for a noninvasive, simple, inexpensive, and easy-to-use diagnostic tool^13^,^14^.

Our study used a gas chromatography-mass spectrometry (GC-MS) method combined with multivariate data analysis to discriminate the VOCs of IgAN, MsPGN and normal samples, to discover potential biomarkers for IgAN and MsPGN in urinary VOCs.

## Methods

### Human Subjects

The present experiments were conducted in accordance with the Declaration of Helsinki^15^. The protocol in this study was approved by the Ethics Committee at the First Affiliated Hospital of Harbin Medical University (No.201314), and written informed consent was obtained from patients prior to study enrollment. This study was conducted between Dec. 2013 and Sep. 2014 at the Department of Anesthesiology and Nephrology in the First Affiliated Hospital of Harbin Medical University.

Included in this study were men between 19 and 42, and women between 24 and 62 years of age identified as ASA I and II individuals and scheduled for renal biopsy. In addition to the group of kidney disease patients, this study also examined healthy volunteers. The following inclusion criteria were utilized for these individuals: 1) negative for kidney diseases, 2) negative for urinary infection, and 3) renal function were normal. This study involved a total of 36 patients with histologically confirmed cases of kidney disease (including 15 individuals with MsPGN, 21 individuals with IgAN) and 15 healthy volunteers. The demographic characteristics are summarized in Table 1.

As detailed in Table 1, the normal control group involved 15 patients. The 15 MsPGN patients who were selected included 5 males and 10 females. The mean age of the MsPGN patients was 37 y, with a standard deviation (SD) of 11.9 y, and 4 of these patients were smokers. There were 8 males and 13 females in 21 IgAN patients were selected. The mean age of the IgAN patients was 36 y, with a SD of 8.0 y, and 6 of these patients were smokers.

### Urine Collection

Portions of midstream urine samples of fasting patients were collected severally in the morning before analysis. All samples were analyzed within 1 h post-sampling.

### Solid-Phase Microextraction(SPME)

A manual SPME holder with carboxen/polydimethylsiloxane (CAR/PDMS) fibers of 75 um thickness was purchased from Supelco (Bellefonte, USA). The SPME fiber was inserted into the vial and exposed to the gaseous sample for 20 min at 40 °C. Subsequently, the desorption of volatiles occurred in the hot GC injector at 200 °C for 2 min.

### GC/MS Analysis

Analysis was performed on a GC/MS (Shimadzu GC-MS QP 2010, Shimadzu, Japan) equipped with a DB-5MS (length 30 m * ID 0.250 * film thickness 0.25 um; Agilent Technologies, USA) plot column. Injections were performed in the splitless mode. The temperature of injector was 200 °C. The flow rate of the helium (99.999%) carrier gas was kept constant at 2 ml min^−1^. The column temperature was held at 40 °C for 1 min to concentrate the hydrocarbons at the head of the column and then increased by 5 °C min^−1^ to 200 °C for 1 min, then ramped 15 °C min^−1^ to 230 °C. The MS analyses were performed in full-scan mode, using a scan range from 35–350 amu. The ion source was maintained at 230 °C, and an ionization energy of 70 eV was used for each measurement.

### Extraction and Pretreatment of the GC/MS Raw Data

Raw GC/MS data were converted into CDF format (NetCDF) files using Shimadzu GCMS Postrun Analysis software and subsequently processed using the XCMS toolbox. The XCMS parameters consisted of the default settings with the following exceptions: xcmsSet (fwhm = 8, snthresh = 6, max = 200); retcor (method = “linear,” family = “gaussian,” plottype = “mdevden”); and a bandwidth of eight for first grouping command and four for the second grouping command. The data set of the aligned mass ions was exported from XCMS and could be further processed using Microsoft Excel to normalize the data prior to multivariate analyses.

### Statistical Analysis

Before statistical analysis, we performed total area normalization for each sample. Then normalized data were exported to SIMCA-P 11.5 platform for principal component analysis(PCA) and orthogonal partial least-squares discriminant analysis (OPLSDA). In order to avoid the occurrence of overfitting, permutation tests with 100 iterations were performed to validate the supervised model. Additionally, in order to determine the significance of each metabolite, the nonparametric Kruskal-Wallis rank sum test was performed. Based on variable importance in the projection (VIP) values calculated from the OPLSDA model and P-values from the nonparametric test, potential metabolic biomarkers were selected using thresholds of 1.2 and 0.05, respectively. Standard measures of accuracy (area under the curve [AUC], sensitivity, specificity) have been computed subsequently by SPSS 13.0.

## Results

### MsPGN and IgAN Patients versus Controls

**Figure 1:**
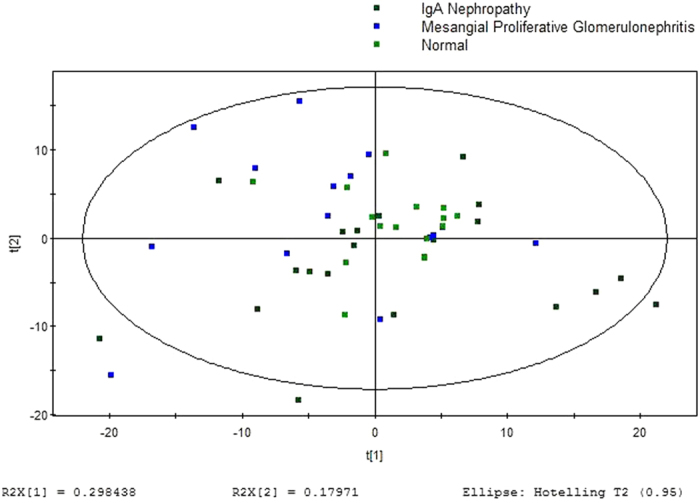
PCA score plot for urine samples from MsPGN and IgAN Patients versus Controls: (7 components, R2X = 0.809, Q2 = 0.590).

**Figure 2:**
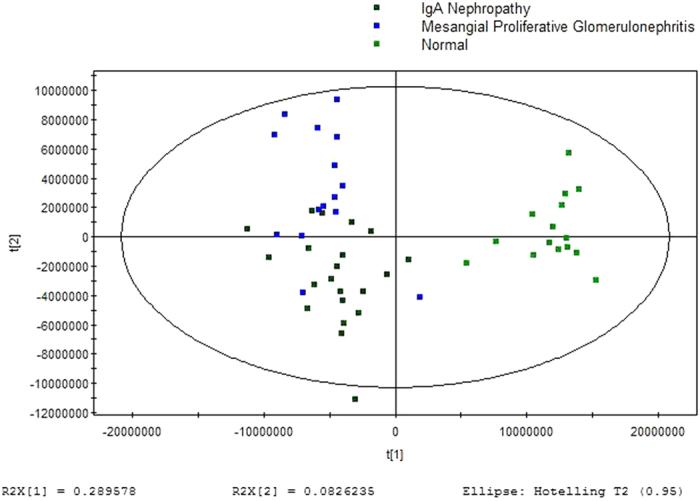
OPLSDA score plot for urine samples from MsPGN and IgAN Patients versus Controls: (4 components, R2X = 0.372, R2Y = 0.631, Q2 = 0.482).

GC/MS was utilized to analyze the metabolites in the urine from 15 MsPGN patients, 21 IgAN patients and 15 healthy controls. Based on the ion peaks in the resulting chromatogram, we obtained 251 variables. The separation trend for the experimental group and the control group was detected from the PCA (Fig. 1) and OPLSDA (Fig. 2) score plots; the tight clustering of samples in the OPLSDA score plot demonstrated that our approach was effective (Fig. 2).

**Figure 3:**
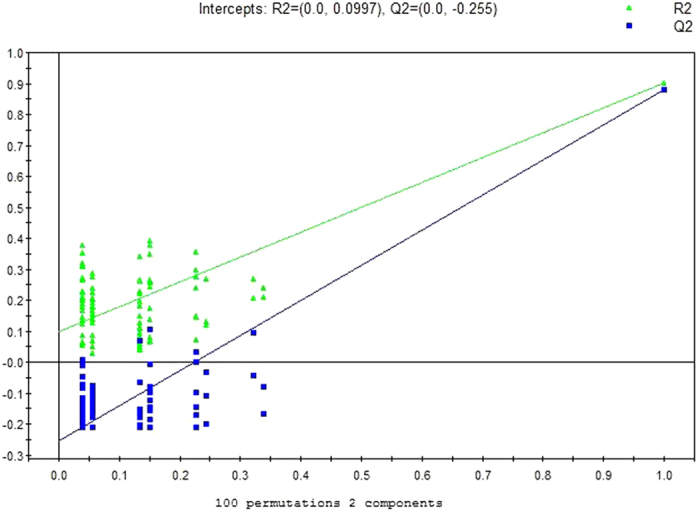
PLSDA validation plot intercepts for urine samples from MsPGN and IgAN Patients versus Controls: R2 = (0.0, 0.0997); Q2 = (0.0, −0.225).

A total of 10 metabolites were consistently detected from the MsPGN patients, IgAN patients and normal controls. Though the two-dimensional PCA score plot displayed a poor separation trend (Fig. 1), the OPLSDA score plot demonstrated a separation among the MsPGN patients, IgAN patients and normal controls using one predictive component and three orthogonal components (R2X = 0.372; R2Y = 0.631; Q2 = 0.482; Fig. 2). Moreover, the R2 and Q2 values calculated from the permutated data were lower than the original values in the validation plot, which confirmed the validity of the supervised model (Fig. 3).

### MsPGN Patients versus IgAN Patients

**Figure 4:**
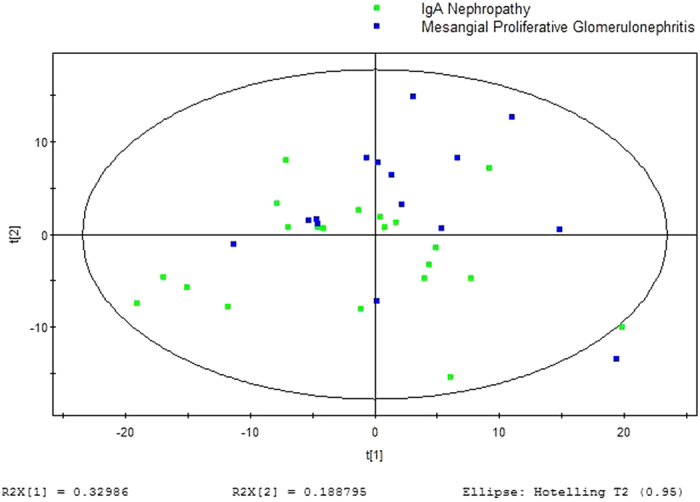
PCA score plot for urine samples from MsPGN versus IgAN Patients: (2 components, R2X = 0.519, Q2 = 0.425).

**Figure 5:**
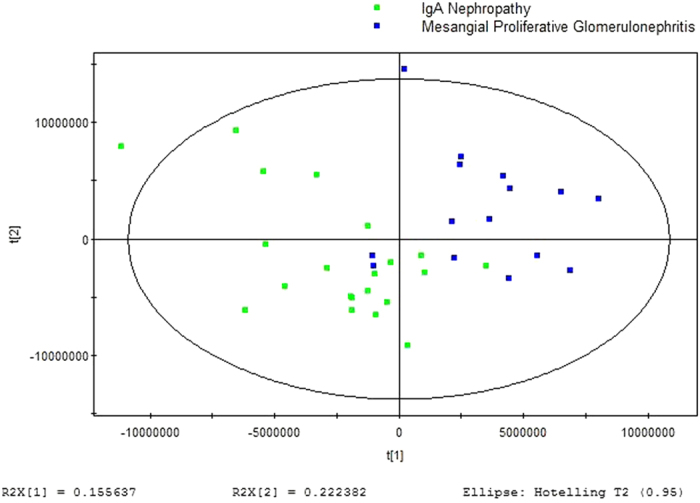
OPLSDA score plot for urine samples from MsPGN versus IgAN Patients: (2 components, R2X = 0.378, R2Y = 0.634, Q2 = 0.127).

GC/MS was utilized to analyze the metabolites in the urine from 15 MsPGN patients and 21 IgAN patients. Based on the ion peaks in the resulting chromatogram, we obtained 248 variables. The separation trend for the experimental group and the control group was detected from the PCA (Fig. 4) and OPLSDA (Fig. 5) score plots; the tight clustering of samples in the OPLSDA score plot demonstrated that our approach was effective (Fig. 5).

**Figure 6:**
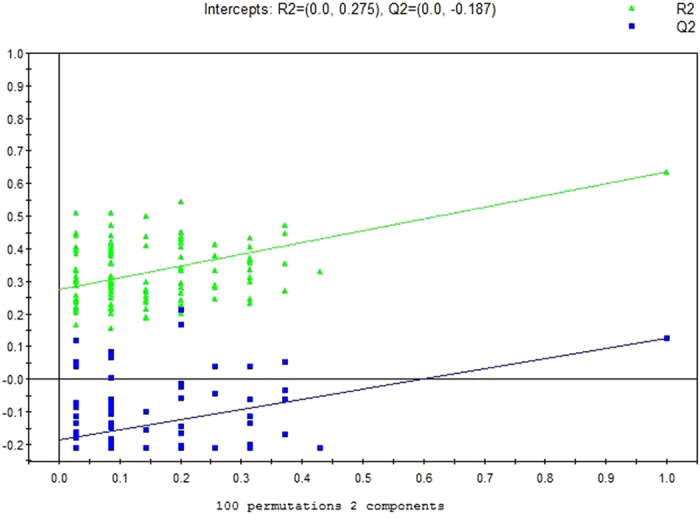
PLSDA validation plot intercepts for urine samples from MsPGN versus IgAN Patients: R2 = (0.0, 0.275); Q2 = (0.0, 0.187).

A total of five metabolites were consistently detected from the MsPGN patients, IgAN patients. Though the two-dimensional PCA score plot displayed a poor separation trend (Fig. 4), the OPLSDA score plot demonstrated a separation between the MsPGN patients and IgAN patients using one predictive component and three orthogonal components (R2X = 0.378; R2Y = 0.634; Q2 = 0.127; Fig. 5). Moreover, the R2 and Q2 values calculated from the permutated data were lower than the original values in the validation plot, which confirmed the validity of the supervised model (Fig. 6).

### Potential Biomarkers

Among the significant metabolites identified using the VIP values in the OPLSDA model and the FDR values, 15 differential metabolites were annotated using the NIST 11 database with a similarity threshold of 72%.

**Figure 7:**
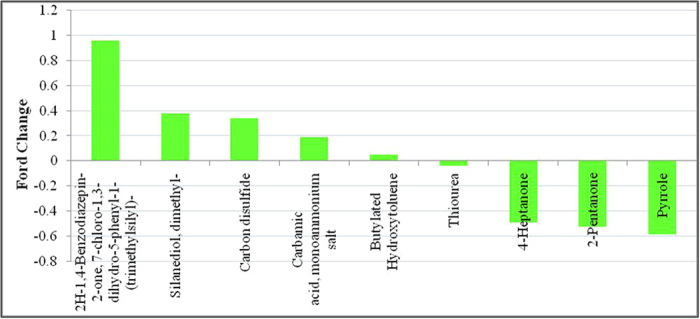
Related metabolites that exist at abnormal levels in the urine from MsPGN patients.

The results showed that, in the experimental group, five metabolites were significantly greater in the group of MsPGN patients than in the normal control group (P < 0.05): Carbamic acid, monoammonium salt; Carbon disulfide; Silanediol, dimethyl-; 2H-1,4-Benzodiazepin-2-one, 7-chloro-1,3-dihydro-5-phenyl-1-(trimethylsilyl)-; Butylated Hydroxytoluene. Moreover, significantly reduced levels of Thioure, 2-Pentanone, Pyrrole and 4-Heptanone were detected in the group of MsPGN patients than in the normal group (P < 0.05, Table 2, Fig. 7).

**Figure 8:**
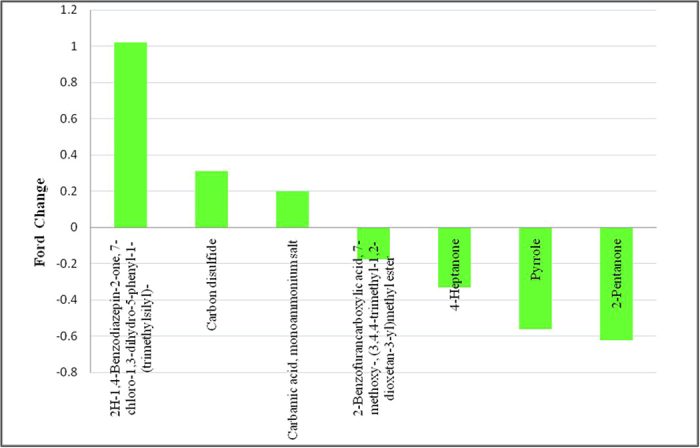
Related metabolites that exist at abnormal levels in the urine from IgAN patients.

Three metabolites were found at increased levels and three at reduced levels in the group of IgAN patients compared with the normal controls, which were of the same metabolites found in the MsPGN patients compared with the normal controls (P < 0.05): Carbamic acid; monoammonium salt; Carbon disulfide and 2H-1,4-Benzodiazepin-2-one,7-chloro-1,3-dihydro-5-phenyl-1-(trimethylsilyl)-; 2-Pentanone; Pyrrole and 4-Heptanone; respectively. In addition, 2-Benzofurancarboxylic acid, 7-methoxy-,(3,4,4-trimethyl-1,2-dioxetan-3-yl) methyl ester exhibited significantly reduced levels in the group of IgAN patients compared with the normal control group (P < 0.05, Table 3, Fig. 8).

**Figure 9:**
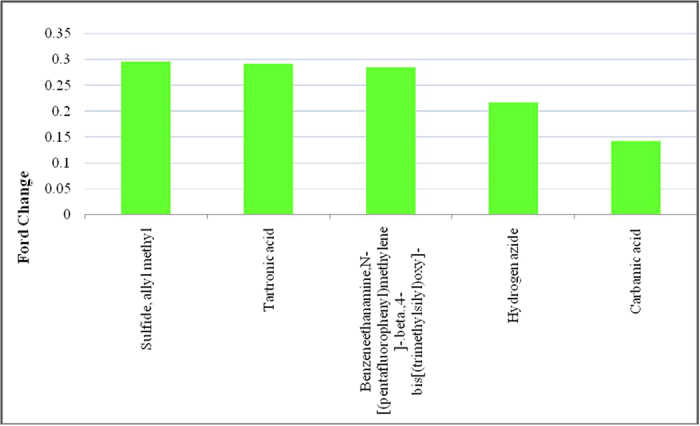
Related metabolites that exist at abnormal levels in the urine between MsPGN patients and IgAN patients.

Five metabolites were significantly increased in the group of IgAN patients compared with the MsPGN patients (P < 0.05): Tartronic acid; Carbamic acid; Sulfide; allyl methyl; Hydrogen azide and Benzeneethanamine,N-[(pentafluorophenyl)methylene]-.beta.,4-bis[(trimethylsilyl)oxy]- (Table 4, Fig. 9). The comparison between the urine samples from the MsPGN patients and IgAN patients revealed that these five metabolites may be specific biomarkers for distinguishing between MsPGN and IgAN.

Metabolic signatures for MsPGN patients vs normal controls resulted in an AUC of 0.196 ~ 0.907. Pyrrole had the largest AUC value which was 0.907, with a sensitivity of 0.933 and a specificity of 0.867 at the best cut-off point. 2-Pentanone had the second largest AUC value which was 0.831, with a sensitivity of 0.933 and a specificity of 0.600 at the best cut-off point.

Metabolic signatures for IgAN patients vs normal controls resulted in an AUC of 0.295 ~ 0.889. Pyrrole had the largest AUC value which was 0.889, with a sensitivity of 0.933 and a specificity of 0.857 at the best cut-off point. 2-Pentanone had the second largest AUC value which was 0.870, with a sensitivity of 0.933 and a specificity of 0.667 at the best cut-off point.

Metabolic signatures for MsPGN patients vs IgAN patients resulted in an AUC of 0.317 ~ 0.552. Sulfide, allyl methyl had the largest AUC value which was 0.552, with a sensitivity of 0.933 and a specificity of 0.810 at the best cut-off point. Tartronic acid had the second largest AUC value which was 0.517, with a sensitivity of 0.933 and a specificity of 0.762 at the best cut-off point.

## Discussion

MsPGN is the main histopathological finding worldwide of glomerulonephritis. The pathogenesis of MsPGN is still unknown, and recent studies suggested that IgA-associated immune complexes are either formed *in situ* or deposited in the mesangium, resulting in a local T-cell mediated inflammatory response, mesangial proliferation and glomerular injury^16^. IgAN is the most common form of primary glomerulonephritis and the major cause of end stage kidney disease worldwide. As an immune complex-mediated disorder, IgAN is immunohistologically defined by the presence of glomerular IgA deposits. The exact pathogenesis of the disease has not been elucidated thus far. Increased production of IgA1 and its defective galactosylation has been considered the inciting event of IgAN onset. Other mechanisms include decreased hepatic clearance of IgA1 and decreased activity of β1, 3-galactosyl transferase that normally adds galactose to IgA1 molecule. Defectively galactosylated IgA deposits in mesangial cells followed by the release of cytokines and growth factors culminates in renal injury^17^. Recently, studies suggested that oxidative stress linked to Gal-deficient IgA1 (GdIgA1) may be involved in the pathogenesis of IgAN^18^. Camilla R *et al*. also found that IgA1 isolated from sera of patients with IgAN contains elevated levels of GdIgA1 within immune complexes, and these complexes stimulate cultured human mesangial cells, resulting in the activation of oxidative stress pathways^19^.

Urine analysis for the routine monitoring of metabolic disorders has attracted a considerable amount of scientific interest, and urinary metabolomic studies have already been applied to many diseases. The potential advantages of urine analysis over other conventional medical tests include its noninvasive nature, low cost and safety. These types of analysis are easy for patients to undergo and can be performed as often as needed. Apart from the possibility of non-invasive diagnostic testing, urine has other advantages over breath, serum or cerebrospinal fluid^20^. Urine is an important reservoir of human scent constituents and has a high abundance of VOCs resulting from the kidneys’ preconcentration capabilities^21^. Many metabolites occur in urine at nearly the same concentrations as the plasma, but the total volatile metabolites in the plasma are relatively low. Until now, more than 230 VOCs belonging to different chemical classes (e.g, aldehydes, ketones, furans, pyrroles, terpenes, and sulfur-containing compounds) have been detected in human urine^21^,^22^,^23^. Thus, the relative enrichment of volatile components makes urine an attractive target for a volatile component metabolomic profiling approach^20^.

In our study, a total of fifteen VOCs were found, ten for discriminating IgAN and MsPGN from normal samples and others for discriminating between IgAN and MsPGN. Several common VOCs included Carbamic acid, mono ammonium salt; Thiourea; Carbon disulfide; 2-Pentanone; Pyrrole; Silanediol, dimethyl-; 4-Heptanone; Butylated Hydroxytoluene; Tartronic acid; Carbamic acid; Sulfide, allyl methyl and hydrogen azide.

Based on their chemical compositions, 2-heptanone and 4-Heptanone are ketones. Their concentrations were decreased in the urine of the IgAN and MsPGN patients matched with the normal controls. Mochalski P *et al*.^24^ reported two potential pathways that could be involved in ketone production: (i) the oxidation of secondary alcohols catalyzed by ADHs and (ii) the *β*-oxidation of branched-chain fatty acids. Hence, 2-pentanone could stem from 2-pentanol, and 2-nonanone could be derived from 2-nonanol. The source of these secondary alcohols remains unclear^24^. Furthermore, several studies showed that 4-heptanone was produced from the plasticizer 2-ethylhexanol and its metabolic intermediate 2-ethylhexanoic acid as a decarboxylation product^25^,^26^.

It is well known that ketones and their metabolites present in human urine. Thus, we suspect these results may due to renal insufficiency in which concentration, absorptive and excretory functions were impaired. The quantitative determination of total 4-heptanone using an extraction and gas chromatographic-mass fragmentographic procedures have demonstrated concentrations of 10–50 nmol/l in normal serum, whereas the concentrations in the serum of patients with chronic renal insufficiency were 10- to 40-fold higher^25^. This finding indicates that in cases of renal insufficiency, the excretion of total 4-heptanone is reduced^25^. This conclusion was consistent with our results.

Urinary pyrroles have endogenous and exogenous origins. The endogenous pyrroles can come from the metabolism of amino sugars and N-acetylneurominic acid in the central nervous system. They can also be a by-product in the synthesis of porphyrins, bile pigment, or an oxidation product of hemopyrrole and bilirubin. Exogenous pyrroles are present in numerous beverages. Nevertheless, the mechanism by which pyrroles are produced in human body and appear in the urine is still unclear^23^.

## Conclusions

Compared with healthy subjects, MsPGN and IgAN have unique VOC profiles, respectively, suggesting that these profiles may be useful as diagnostic assays for MsPGN and IgAN.

## Additional Information

**How to cite this article**: Feng, Y. *et al*. Volatile Organic Metabolites Identify Patients with Mesangial Proliferative Glomerulonephritis, IgA Nephropathy and Normal Controls. *Sci. Rep*. **5**, 14744; doi: 10.1038/srep14744 (2015).

## Figures and Tables

**Table 1:** Demographic characteristics of the study subjects.

	Normal	MsPGN	IgAN
subjects(n)	15	15	21
age	35(7.6)	37(11.9)	36(8.0)
male	7	5	8
female	8	10	13
smokers(n)	3	4	6
SCR(umol/L)		94.1(45.1)	121.9(92.1)
ALB(g)		33.7(7.3)	36.9(6.1)
24h urine protein(g)		2.7(2.0)	2.2(1.7)

Abbreviations: SCR, serum creatinine; ALB, plasma albumin.

**Table 2:** Related metabolites that exist at abnormal levels in the urine from MsPGN patients.

potential biomarkers	RT	VIP	P	FC
2H-1,4-Benzodiazepin-2-one, 7-chloro-1,3-dihydro-5-phenyl-1-(trimethylsilyl)-	17.8	1.65659	0.003453	0.954461
Silanediol, dimethyl-	3.613197	1.77836	0.004494	0.377826
Carbon disulfide	1.475	12.5682	3.75E-06	0.337784
Carbamic acid, monoammonium salt	1.141667	3.78809	0.000125	0.187348
Butylated Hydroxytoluene	22.94167	1.43036	0.002001	0.051647
Thiourea	1.35	1.67009	0.00581	−0.03801
4-Heptanone	5.475	2.89244	0.000125	−0.48707
2-Pentanone	2.3	4.78673	8.87E-05	−0.52189
Pyrrole	3.075	2.76819	0.000125	−0.58163

Abbreviations: RT, retention time; VIP, variable importance in the projection; FC, fold change, defined as: FC = log10(X2/X1), while X1 denoted the arithmetic mean value of certain metabolite in the control group and X2 denoted the arithmetic mean value in the case group. FC with a positive value indicates that the concentration of certain metabolite is relatively higher in MsPGN patients compared with healthy controls. FC with a negative value indicates that the concentration of certain metabolite is relatively lower in MsPGN patients compared with healthy controls.

**Table 3:** Related metabolites that exist at abnormal levels in the urine from IgAN patients.

potential biomarkers	RT	VIP	P	FC
2H-1,4-Benzodiazepin-2-one, 7-chloro-1,3-dihydro-5-phenyl-1-(trimethylsilyl)-	17.8	2.06063	0.001257	1.021583
Carbon disulfide	1.475	11.7166	2.82E-05	0.309181
Carbamic acid, monoammonium salt	1.141667	4.30661	0.000711	0.198104
2-Benzofurancarboxylic acid, 7-methoxy-, (3,4,4-trimethyl-1,2-dioxetan-3-yl)methyl ester	1.35	2.64804	0.000126	−0.17541
4-Heptanone	5.475	2.54699	0.000442	−0.33223
Pyrrole	3.075	2.88632	8.47E-05	−0.56124
2-Pentanone	2.3	5.40358	0.000347	−0.62353

Abbreviations: RT, retention time; VIP, variable importance in the projection; FC, fold change, defined as: FC = log10(X2/X1), while X1 denoted the arithmetic mean value of certain metabolite in the control group and X2 denoted the arithmetic mean value in the case group. FC with a positive value indicates that the concentration of certain metabolite is relatively higher in IgAN patients compared with healthy controls. FC with a negative value indicates that the concentration of certain metabolite is relatively lower in IgAN patients compared with healthy controls.

**Table 4:** Related metabolites that exist at abnormal levels in the urine between MsPGN patients and IgAN patients.

potential biomarkers	RT	VIP	P	FC
Sulfide, allyl methyl	2.333333	1.27078	0.025743	0.295425
Tartronic acid	1.158333	1.393	0.014101	0.291611
Benzeneethanamine,N-[(pentafluorophenyl)methylene]-.beta.,4-bis[(trimethylsilyl)oxy]-	13.05833	1.94748	0.030319	0.28431
Hydrogen azide	3.05	2.32053	0.030319	0.217254
Carbamic acid	1.991667	1.57673	0.011774	0.142994

Abbreviations: RT, retention time; VIP, variable importance in the projection; FC, fold change, defined as: FC = log10(X2/X1), while X1 denoted the arithmetic mean value of certain metabolite in the MsPGN patients group and X2 denoted the arithmetic mean value in the IgAN patients group. FC with a positive value indicates that the concentration of certain metabolite is relatively higher in IgAN patients compared with MsPGN patients.
